# Natural *Plasmodium* infection in monkeys in the state of Rondônia (Brazilian Western Amazon)

**DOI:** 10.1186/1475-2875-12-180

**Published:** 2013-06-03

**Authors:** Maisa S Araújo, Mariluce R Messias, Marivaldo R Figueiró, Luiz Herman S Gil, Christian M Probst, Newton M Vidal, Tony H Katsuragawa, Marco A Krieger, Luiz H Pereira da Silva, Luiz S Ozaki

**Affiliations:** 1 Fundação Oswaldo Cruz, Fiocruz Rondônia/Instituto de Pesquisa em Patologias Tropicais (IPEPATRO), Fiocruz Rondônia, Rua da Beira 7671, Bairro Lagoa, Porto Velho RO CEP 76812-245, Brazil; 2 Departamento de Biologia, Universidade Federal de Rondônia, BR 364, Campus José Ribeiro Filho, Porto Velho RO CEP: 78900000, Brazil; 3 Embrapa Amazônia Oriental, Travessa Dr. Eneas Pinheiro, PO Box 48, Belém PA CEP 66095-100, Brazil; 4 Instituto Carlos Chagas, Curitiba – Fiocruz, Algacyr Munhoz Mader, 3775, Curitiba, PR CIC 81350-010, Brazil; 5 Center for the Study of Biological Complexity (CSBC), Life Sciences, Virginia Commonwealth University, Grace E. Harris Hall, Room 3131, 1015 Floyd Ave, Richmond, VA, 23284, USA; 6 Department of Microbiology and Immunology, 1101 E Marshall St, Sanger Hall 5-036, Richmond, VA 23298, USA

**Keywords:** Simian malaria, New World monkeys, *Plasmodium*, Amazonian forest, 18S rRNA

## Abstract

**Background:**

Simian malaria is still an open question concerning the species of *Plasmodium* parasites and species of New World monkeys susceptible to the parasites. In addition, the lingering question as to whether these animals are reservoirs for human malaria might become important especially in a scenario of eradication of the disease. To aid in the answers to these questions, monkeys were surveyed for malaria parasite natural infection in the Amazonian state of Rondônia, Brazil, a state with intense environmental alterations due to human activities, which facilitated sampling of the animals.

**Methods:**

Parasites were detected and identified in DNA from blood of monkeys, by PCR with primers for the 18S rRNA, CSP and MSP1 genes and sequencing of the amplified fragments. Multiplex PCR primers for the 18S rRNA genes were designed for the parasite species *Plasmodium falciparum* and *Plasmodium vivax, Plasmodium malariae/Plasmodium brasilianum* and *Plasmodium simium*.

**Results:**

An overall infection rate of 10.9% was observed or 20 out 184 monkey specimens surveyed, mostly by *P. brasilianum*. However, four specimens of monkeys were found infected with *P. falciparum*, two of them doubly infected with *P. brasilianum* and *P. falciparum*. In addition, a species of monkey of the family Aotidae, *Aotus nigriceps*, is firstly reported here naturally infected with *P. brasilianum*. None of the monkeys surveyed was found infected with *P. simium*/*P. vivax*.

**Conclusion:**

The rate of natural *Plasmodium* infection in monkeys in the Brazilian state of Rondônia is in line with previous surveys of simian malaria in the Amazon region. The fact that a monkey species was found that had not previously been described to harbour malaria parasites indicates that the list of monkey species susceptible to *Plasmodium* infection is yet to be completed. Furthermore, finding monkeys in the region infected with *P. falciparum* clearly indicates parasite transfer from humans to the animals. Whether this parasite can be transferred back to humans and how persistent the parasite is in monkeys in the wild so to be efficient reservoirs of the disease, is yet to be evaluated. Finding different species of monkeys infected with this parasite species suggests indeed that these animals can act as reservoirs of human malaria.

## Background

Malaria is a mosquito-borne disease of humans and other vertebrates caused by protozoa of the genus *Plasmodium*. Approximately 200 species of mammals, reptiles and birds are potential host to the parasite. Primates represent more than half of the host species [1]. Currently, 33 parasite species are recognized as simian malaria, occurring in multiple species of prosimians, New World, and Old World monkeys, African and Asian apes [2-5].

With the most diverse simian fauna in the world, only two species of simian malaria have been described in Brazil: *Plasmodium brasilianum,* a quartan malaria first described by Gonder and Berenberg-Gossler [6] in bald uakari (*Cacajao calvus*) in the upper Amazon (northern Brazil) and *Plasmodium simium*, a tertian malaria, described by Fonseca [7] in a howler monkey (*Alouatta fusca*) in the State of São Paulo (southern Brazil).

*Plasmodium brasilianum* has the widest host range. It has been described infecting 35 species of monkeys mostly Cebidae and Atelidae, and rarely in members of the family Callithricidae [8]. In contrast, *P. simium* has been described in only three primate species: *Alouatta guariba, Brachyteles arachnoids*[9] and *Alouatta caraya*[10,11]. Geographic distribution of hosts infected by *P. simium* is restricted to the Atlantic forest (southern Brazil) while *P. brasilianum* infected hosts have been described in Brazil, Panama, Venezuela, Peru and Colombia [12,13].

*Plasmodium simium* was never described in other countries or even in northern Brazil [14]. However, the lists of both susceptible monkey species and malaria parasite species themselves in the Amazon forest might be incomplete. The main reason for this underestimation is probably due to the extent of the forests and the difficulties derived thereof in obtaining monkey samples. Supporting this hypothesis are results of the present survey of simian malaria in areas of the Brazilian state of Rondônia and bordering states. For instance, in this survey a species of monkey, *Aotus nigriceps*, infected with *P. brasilianum*, is firstly described here. Furthermore, found in this survey were four species (*Alouatta puruensis, Lagothrix cana cana, Ateles chamek* and *Callicebus brunneus*) that was positive for *Plasmodium falciparum* in PCR assays. Description of New World monkeys infected by *P. falciparum* has been previously reported in *Alouatta guariba* and *Alouatta caraya* in the Brazilian state of São Paulo [11].

Reported here are findings of *Plasmodium* infection in a total of 184 monkeys surveyed in the Brazilian state of Rondônia. Blood samples were analysed from animals: a) kept in captivity, and animals found in the wild, broadly described as b) animals in remnants of forests in areas with present environmental instability (hydroelectric dam construction, recently deforested for wood extraction, etc.) and, c) in remnants of forests in areas with past history of environmental instability (past mining and rubber extraction, land opened for agricultural activities, etc.). For the detection and identification of *Plasmodium* species polymerase chain reaction (PCR) and partial sequencing of parasite 18S rRNA, circumsporozoite protein (CSP) and merozoite surface protein 1 (MSP1) genes were used.

## Methods

### Origin of monkey specimens

Blood samples were collected from a total of 184 monkeys, originating as follows: a) monkeys kept in captivity. These are animals kept in captivity and close to urban areas, i.e., at Porto Velho Ecological Park and facilities of the IBAMA (Brazilian Institute of Environment and Renewable Natural Resources) in Rondônia. A total of 49 captive monkeys were sampled. Monkeys in the wild were sampled in remnants of forests close to areas: b) with present environmental instability, i.e., with wood extraction (Manoa Farm in Cujubim), hydroelectric plant construction sites (Jirau and Santo Antônio at the Madeira river), another hydroelectric plant (Rondon II in Pimenta Bueno), and two recent land occupation sites (São Francisco do Canutama, and Candeias do Jamari). A total of 82 samples were collected at these areas; and, c) areas with past history of environmental instability, i.e., past tin mining (Flona do Jamari in Itapuã), rubber extraction (Machadinho D’Oeste and Costa Marques), and two sites presently exploited for agriculture (Cabixi and Comodoro). A total of 53 samples were collected at these areas.

### PCR primers design and sensibility testing

Parasite detection and species identification was performed using primers specific for the 18S rRNA gene of *P. falciparum*, *Plasmodium vivax*, *Plasmodium malariae/P. brasilianum* and *P. simium*, in a semi-nested multiplex PCR (Table 1). Primers for the 18S rRNA genes of the various *Plasmodium* species were designed by comparing the gene sequences deposited at NCBI GenBank using the program Sequencher® (version 5.1, Gene Codes Corporation, Ann Arbor, MI USA). The sensibility of the PCR with the designed primers was verified as follows. For *P. falciparum*, a solution of DNA of cultured *P. falciparum* 3D7 cells prepared with a DNA extraction kit (Illustra blood genomicPrep Mini Spin Kit, GE Health Care), was 10 fold serially diluted down to sub-picogram (pg) level (0.025 pg). Five microliters of the10 fold serially diluted DNA solutions were added to a final volume of 15 μl of PCR mixtures. The threshold detection limit in this assay was about 0.125 pg of parasite DNA. This amount of DNA is equivalent to about 5 copies of the *P. falciparum* genome assuming a genome size of 23 megabases (Mb) [15] or 4.6×10^7^ base pairs (bp). As the volume of DNA added to the PCR (5 μl) is equivalent to about 10 μl of blood and assuming that 1 μl of blood contains about 5×10^6^ red blood cells (RBC), and one single genome per infected RBC, the parasitaemia equivalent to this amount of DNA is therefore 0.00001%. For calculating the detection threshold of *P. vivax,* a DNA from a vivax malaria patient with the buffy coat removed was used. Similarly, the calculated detection threshold for *P. vivax* DNA was 500 genomes of 30 Mb size [16] or about 0.001% of parasitaemia. The apparent lower sensitivity of the PCR for *P. vivax* DNA might be due to contaminating host DNA, which contamination level have not yet been determined. For *P. brasilianum*, the DNA of a positive monkey blood, prepared similarly to the *P. vivax* DNA, was used and the calculated detection threshold was in the range of 0.001-0.003%. For this calculation the genome of *P. brasilianum* was assumed to be of 30 Mb.

**Table 1:** **PCR primers used for the detection and identification of *Plasmodium *sp**

**Primer**	**Sequence**	**Ref.**
1^st^ PCR		This work
plgen18SF	TATTAAAATTGTTGCAGTTAAARCG	
plgen18SR	ATCTGTCAATCCTACTCTTGTC	
2^nd^ PCR^a^		This work
fal18SF	GAATCCGATGTTTCATTTAAACTGG	
viv18SF	TAACGCCGTTAGCTAGATCCAC	
bra18SF	GTTAAAACAGTTTCTGTGTTTGAATA	
sim18SF	AGATTTTCTGGAGACAAACAACTGC	
CSP^b^		This work
mbraCSPF	CATGAAGAAGTTATTCTGTCTTAGC	
mbraCSPR	TTAGTGAAAGAGTATTAAGACTAAAAC	
MSP-1^b^		This work
mbraMSP1F	ATGAAGATTATGAACAACTTATTCAAAAG	
mbraMSP1R	CATCTGACCCTGATTCACTAGG	

^a^ PCR primers designed for the 18S rRNA genes of *Plasmodium* species observed in South America (*P. falciparum, P. vivax, P. malariae/brasilianum and P. simium*) and based on sequences deposited in GenBank.

^b^ Primers based on CSP and MSP1 sequences of *P. malariae*/*P. brasilianum* deposited in GenBank.

### Blood sampling and parasitological tests

Blood was drawn by femoral vein puncture of monkeys previously anesthetized with proportional doses of Diazepam [17] when necessary, and samples collected in vacuum tubes containing citrate solution (BD Indústrias Cirúrgicas, Ltda, São Paulo, SP, Brazil). Two thick and two thin smears were prepared from each blood sample, Giemsa-stained and microscopically analysed by standard procedures [18]. Blood samples were transported to the laboratory on ice, fractionated by centrifugation into red/white blood cells and plasma. The fractionated samples were frozen at −20°C until further analyses.

DNA from 100 μl of the red blood cell fraction (equivalent to about 200 μl of whole blood) was purified with a DNA extraction Kit (Illustra blood genomicPrep Mini Spin Kit, GE Health Care). The PCR mixture for the first reaction contained: HotMaster Taq (5 PRIME, Inc., Gaithersburg, MD, USA) buffer (10X taq buffer with 25 mM mg^2+^), 200 μM of each dNTP, the PCR primers (0.25 μM each primer plgen18SF and plgen18SR), 0.3 units of HotMaster Taq polymerase and 5 μl of template DNA in a final volume of 15 μl. In the multiplex PCR, the reaction mixture contained: HotMaster Taq buffer (10X taq buffer with 25 mM mg^2+^), 200 μM of each dNTP, the PCR primers (0.25 μM each primer plgen18SR, fal18SF, viv18SF, mal/bra18SF, sim18SF), 0.3 units of HotMaster Taq polymerase and 1.5 μl of the product the first reaction the same volume of the first reaction.

The PCR was performed in a Veriti 96 Well Thermal Cycler (Applied Biosystems, Life Technologies) under the following conditions: first reaction - denaturation at 95°C for 3 min, followed by 30 cycles at 95°C for 3 sec, 52°C for 30 sec and 68°C for 1 min; second reaction (multiplex) - denaturation at 95°C for 3 min, followed by 30 cycles at 95°C for 30 sec, 55°C for 30 sec and 68°C for 1 min. The amplification products (3 μl) were run on 1% agarose gels in TAE buffer. For all samples positive for *Plasmodium*, DNA extractions and PCR reaction were repeated. All DNA and PCR manipulations were in a laboratory with no other *Plasmodium* source but the monkey samples.

Final identification of *Plasmodium* species was obtained by sequencing of products of the first PCR or second PCR. These were first cloned in pGEM®-T Easy (Promega Co., Madison, WI, USA), the insert amplified by colony PCR using the surrounding plasmid primers SP6/T7 and the amplified fragments sequenced by the di-deoxy method. The raw sequence data were edited and analysed with the program Sequencher® and the sequences identified by BLAST at NCBI/Genbank nr database. One identified sequence of each gene (18S rRNA, CSP and MSP-1) from each monkey specimen was randomly selected and deposited at NCBI GenBank (Accession numbers: KC906706-32).

### Ethical considerations

Approval of the study protocol was obtained from the Animal Ethics Committee of the Research Institute of Tropical Pathology Rondônia, Porto Velho, RO, Brazil (CEUA/IPEPATRO 2009/1). All procedures adopted in this study were approved in full compliance with specific federal guidelines issued by the Brazilian Ministry of Environment (SISBIO, process number 14081–3 and 17302–1).

## Results

### Monkey specimens

Blood samples from a total of 184 monkey specimens were collected and analysed for *Plasmodium* sp by microscopy and partial sequencing of the 18S rRNA gene amplified by PCR. The surveyed monkeys were of the families Callitrichidae, Cebidae, Aotidae, Pitheciidae and Atelidae in a total of 21 species. The most numerous specimens collected were from monkeys of the species *Sapajus apella* (tufted capuchin) (33 specimens) and the least were from the species *Sapajus cay* (headed capuchin), *Callibela humilis* (dwarf marmoset), *Saguinus labiatus* (red bellied tamarin) and *Callicebus bernhardi* (titi monkey), one of each species.

**Figure 1:**
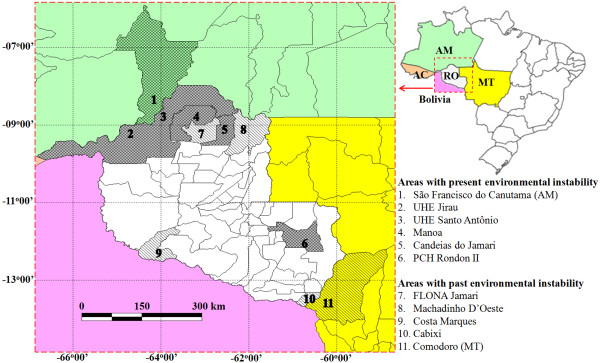
**Monkey survey areas in Rondônia (RO) and bordering states.** Included in the study are bordering areas in the states of Amazon (AM, area 1) and Mato Grosso (MT, area 11). Maps and geographical coordinates were obtained using the software GPS Track Maker PRO (Geo Studio Technology, Brazil) and the images edited with Microsoft Office Powerpoint (Microsoft Co) and Photo-Paint 7 (Corel Co). UHE = Usina Hidrelétrica (Hydroelectric Plant); PCH = Pequena Central Hidrelétrica (Small Hydropower Core); FLONA = Floresta Nacional (National Forest).

Of the184 animals surveyed, 49 were monkeys kept in captivity (10 species) and 135 were monkeys captured in the wild (21 species). Practically, all known species of monkeys in the region were examined except for two species, *Mico rondoni* and *Callimico goeldii* of the family Callitrichidae. Table 2 lists all known species of monkeys found in the region. Monkeys in captivity were animals seized by the IBAMA and kept at the Ecological Park of the city of Porto Velho or facilities of IBAMA in Rondônia. Animals from the wild were from areas with present or past environmental instability (see Figure 1 and Methods). Eighty-two (82) specimens were collected in the first area and 53 in the second.

**Table 2:** ***Plasmodium sp. *infection in non-human primates in Rondônia, Brazil, 2009–2012**

	**Number of specimens**				**Infected**			
**Species**	**Wild**		**Captive**	**Total**	**Wild**		**Captive**	**Total (%)**
	**Unstable**^**a**^	**Stable**^**b**^			**Unstable (%)**^**a**^	**Stable (%)**^**b**^		
**Family callitrichidae**								
*Callibela humilis*	-	-	1	1	-	-	-	-
*Callibela goeldii*	-	-	-	-	-	-	-	-
*Cebuella pygmaea*	4	-	-	4	-	-	-	-
*Mico melanurus*	3	-	-	3	-	-	-	-
*Mico rondoni*	-	-	-	-	-	-	-	-
*Saguinus weddelli*	3	3	-	6	-	-	-	-
*Saguinus labiatus*	1	-	-	1	-	-	-	-
**Family cebidae**								
*Cebus albifrons*	1	-	2	3		-	-	-
*Sapajus apella*	11	6	16	33	1^B^	-	1^B^	2(6.1)
*Sapajus cay*	-	1	-	1	-	-	-	-
*Saimiri boliviensis*	2	-	2	4	-	-	-	-
*Saimiri ustus*	6	6	-	12	1^B^	-	-	1(8.3)
**Family aotidae**								
*Aotus nigriceps*	9	3	2	14	1^B^	-	-	1(7.1)
**Family pitheciidae**								
*Callicebus brunneus*	6	3	2	11	-	1^FB^	-	1(9.1)
*Callicebus dubius*	2	-	-	2	1^B^	-	-	1(50)
*Callicebus bernhardi*	-	1	-	1		-	-	-
*Callicebus cinerascens*	4	2	-	6		-	-	-
*Chiropotes albinasus*	1	1	-	2		1^B^	-	1(50)
*Pithecia irrorata*	13	5	4	22	5^B^	1^B^	-	6(27.3)
**Family atelidae**								
*Alouatta puruensis*	7	7	2	16		-	1^F^	1(6.3)
*Alouatta caraya*	-	4	-	4		-	-	-
*Ateles chamek*	4	9	9	22		3^B^	1^F^	3(13.6)
*Lagothrix cana cana*	5	2	9	16	1^FB^	1^B^	-	2(12.5)
**Total**	**82**	**53**	**49**	**184**	**10(12.2)**	**7(13.2)**	**3(6.1)**	**20(10.9)**

^a^ Specimens from environmentally unstable areas.

^b^ Specimens from environmentally stable areas.

^B^ Positive for *P. brasilianum* only.

^F^ Positive for *P. falciparum* only.

^FB^ Positive for both *P. falciparum* and *P. brasilianum.*

### Parasitological analysis of blood samples

Blood samples were first examined by microscopy of Giemsa-stained thin blood films. PCR and sequencing however identified the parasite as *P. brasilianum*. Almost in all cases, the very low parasitaemia and poor quality of the blood films obtainable in the wild prevented unambiguously identification of parasite species by microscopy. Therefore, identification was carried out entirely by PCR assays and sequencing of portions of parasite18S rRNA gene using *Plasmodium* species specific primers (Table 1) in a semi-nested PCR (see Methods). *P. brasilianum* was detected in 18 of the animals (Table 2). The identity of the parasite as *P. brasilianum* was also confirmed by amplification and sequencing of its MSP-1 and CSP genes. *P. falciparum* was also detected in four monkeys and in two cases as mixed infections (*P. falciparum* plus *P. brasilianum*) (Table 2). Curiously, no *P. vivax* (or *P. simium*) was found infecting any of the 184 monkey specimens in spite of the prevalence of *P. vivax* in the human population in the region. The absence of *P. simium* in northern Brazil has been previously reported by Deane [13], as mentioned above.

In summary, by microscopy parasite infection was detected in only three of the animals while by PCR and sequencing detection was possible in 20 out of the 184 (10.9%) animals surveyed (Table 2).

### Monkey species susceptible to *Plasmodium* infection

The following species of monkeys were found infected with *Plasmodium* spp: *Sapajus apella* and *Saimiri ustus* of the family Cebidae; *Callicebus brunneus*, *Callicebus dubius, Chiropotes albinasus* and *Pithecia irrorata* of the family Pitheciidae; *Ateles chamek* and *Lagothrix cana cana* of the family Atelidae; and *Aotus nigriceps* of the family Aotidae (Table 2). The latter is described here for the first time as being naturally infected with *Plasmodium*, in this case with *P. brasilianum*. Infection by *P. falciparum* was detected in specimens of *Callicebus brunneus*, *Alouatta puruensis, Ateles chamek* and *Lagothrix cana cana* (Table 2) of which two species, *Callicebus brunneus* and *Lagothrix cana cana*, were infected with *P. falciparum* plus *P. brasilianum*. Of note is the lower rate of parasite infection observed in animals kept in captivity (6.1%) as opposed to animals in the wild (12.6%). Due to the low number of specimens examined and the difference in the composition of monkey species in the two sets, weather this different rate of infection is real remains to be further assessed.

## Discussion

The prevalence of *P. brasilianum* in the Amazon region has been reported previously [8,13,19,20]. However, very few epidemiological studies on simian malaria have been performed in Rondônia state in the Brazilian Western Amazon. Current estimates of parasite prevalence and the extant parasite species in circulation as well as the species of primates susceptible to infection by *Plasmodium*, might not be accurate. The study of prevalence of *Plasmodium* parasites in monkeys in the Amazon region is hampered primarily by the difficulties in obtaining samples from the animals and the extension of the forest. In this study investigation was carried out on the occurrence of *Plasmodium* sp infection in monkeys in the state of Rondônia where large perturbations in its forests (high levels of deforestation for agriculture, wood extraction, tin mining and dam construction), facilitated the collection of samples.

In this study, 21 species of monkeys were analysed, practically all known species in the region (Table 2). Of these, specimens of 10 species were found infected with *Plasmodia*, belonging to four out of five families of the extant monkeys in the region, or monkeys of the families Cebidae, Atelidae, Pitheciidae and Aotidae. No specimens of the family Callitrichidae were found infected. *Plasmodium* infection in monkeys of this family is unusual but has been observed especially in areas with environmental instability (construction site of hydroelectric dams) [21,22]. *P. brasilianum* is well known in several species of monkeys, mostly of the families Cebidae and Atelidae, and rarely in individuals from the family Callithricidae [8].

Monkeys of the family Aotidae has been suggested and widely used as model for development and testing of vaccines and drugs against malaria [23,24]. In spite of being the most used animals in experimental malaria infection due to their susceptibility to human plasmodia, species of this family have been consistently found uninfected in previous surveys for simian parasites. A specimen of *Aotus nigriceps* is reported here for the first time as being naturally infected with *Plasmodium*, in this case *P. brasilianum*. This specimen is one out of nine captured in forest remnants of an environmentally unstable area (hydroelectric dam construction Jirau). The only species of this family (Aotidae) which had previously been reported naturally-infected with *Plasmodium* is *Aotus vociferans* from Peru (Peruvian isolate I/CDC strain of *P. brasilianum*) [25].

Four animals were found infected with *P. falciparum*, one of the species *Callicebus brunneus* (family Pitheciidae), one *Lagothrix cana cana*, one *Ateles chamek* and one *Alouatta puruensis* (the latter three are all of the family Atelidae). The first two animals were found doubly infected (*P. brasilianum* and *P. falciparum*) and the latter two showed single infection by *P. falciparum*. Confirmation of this parasite in monkeys in the Amazon region clearly shows that transfer of *P. falciparum* occurs from humans to the animals and might be a frequent event. Whether parasite transfer can occur in the opposite direction, i.e., from the animals back to humans is being assessed. A parameter also necessary to assess is if once the parasite infects a monkey it stably stays in the wild, i.e., if the parasite continues to be transmitted from monkeys to monkeys. Because the parasite is able to complete the cycle in monkeys, as demonstrated experimentally in monkeys of the family Aotidae [26,27] and Cebidae [28,29], it is likely that the monkeys found naturally infected by *P. falciparum* are able to infect mosquitoes and hence maintain the parasite in the wild and eventually back to humans. This type of infection might be frequent as suggested [30] and the possibility that New World monkeys being reservoirs of falciparum malaria as well as of other malarias, cannot thus be discarded.

In spite of *P. vivax* being prevalent in the region, no animal was found infected with the equivalent *P. simium*, a fact previously reported for the Amazon region [13]. This parasite species has been reported infecting only three species of monkeys in the Brazilian Atlantic Forest [8,13]. Of the three species of monkeys found infected by *P. simium*, one is present in the Amazon region, *Alouatta caraya*, species recently found described infected in the Atlantic Forest [11]. The mosquito of the species *Anopheles cruzii* has been incriminated in the transmission of simian malaria in Brazil, which is found infected by *P. brasilianum* and *P. simium*[31]. In recent surveys other mosquito species were found naturally infected with these parasites [32]. The mosquito *An. cruzii* is absent in the Amazon region and the vector that transmits *P. brasilianum*, the most common parasite infecting monkeys in the region, is yet to be clearly determined. Lourenço-de-Oliveira and Luz [33] suggested five vectors, *Anopheles mediopunctatus, Anopheles nuneztovari, Anopheles oswaldoi, Anopheles triannulatus* and *Anopheles shannoni*. They are the most abundant, sylvatic and are blood feeders in the canopy of trees. Mosquito of the species *Anopheles darlingi*, the most important vector of human malaria in the region due to its anthropophilic behaviour and susceptibility to human *Plasmodium*[34,35], has apparently no relation to simian malaria transmission [33]. Other studies however, suggest that this mosquito species can bite not only near the ground but also in the canopy of trees [36] where it could in consequence transmit parasites from humans to monkeys and *vice-versa*. *Anopheles* density tends to change in unstable environment and some vectors may change behaviour, from being primarily zoophilic to being anthrophilic, in adaptation to new environmental conditions [37].

In conclusion, environmental change, directly or indirectly caused by humans, might have in consequence a widening range of hosts to infectious diseases. If malaria is to be controlled or even eradicated, the appearance of alternative host carriers needs special attention. Malaria resurgence has occurred in 91% of the countries in which malaria was under control since the end in 1969 of the Global Malaria Eradication Programme (GMEP). The main cause of the resurgences has been linked to weakening of control measures [38]. In weak malaria control scenarios, in areas such as that described in this report, the presence of animals carrying malaria parasites is a potential source for malaria resurgence. Again, the question of whether the parasites, especially *P. falciparum*, can be perpetuated in the wild by monkey-to-monkey transmission, a condition essential for their role as reservoir of the disease, remains to be answered.

## Conclusions

A molecular epidemiological survey of malaria parasites in captive and wild monkeys was carried out in the state of Rondônia in the Brazilian Western Amazon. It is reported here for the first time a monkey species of the family Aotidae, *Aotus nigriceps*, naturally infected with *Plasmodium* parasite, in this case with *P. brasilianum*. Monkeys infected with the parasite species *P. falciparum* are also firstly described in the region. These new findings point to an incomplete list of New World monkey species susceptible to *Plasmodium* parasites and reinforce the lingering question of whether these animals serve as a reservoir of human malaria.

## Abbreviations

PCR: Polymerase chain reaction; CSP: Circumsporozoite protein; MSP1: Merozoite surface protein 1.

## Competing interests

The authors declare that they have no competing interests.

## Authors’ contributions

LSO and LHPS designed the research, MSA executed the molecular experiments, MRM and MRF coordinated the capture and identification of the animals, LHGS, CMP, NMV, MAK and THK contributed with biological materials, reagents and analytical tools. MSA, LHPS and LSO analysed the data and wrote the manuscript. All authors contributed with comments to the manuscript. All authors read and approved the final manuscript.
